# Recent developments in the chemistry of non-trigonal pnictogen pincer compounds: from bonding to catalysis

**DOI:** 10.1039/d0sc03819a

**Published:** 2020-08-18

**Authors:** Josh Abbenseth, Jose M. Goicoechea

**Affiliations:** Department of Chemistry, Chemistry Research Laboratory, University of Oxford Mansfield Road Oxford OX1 3TA UK Josh.Abbenseth@chem.ox.ac.uk Jose.Goicoechea@chem.ox.ac.uk

## Abstract

The combination of well-established meridionally coordinating, tridentate pincer ligands with group 15 elements affords geometrically constrained non-trigonal pnictogen pincer compounds. These species show remarkable activity in challenging element–hydrogen bond scission reactions, such as the activation of ammonia. The electronic structures of these compounds and the implications they have on their electrochemical properties and transition metal coordination are described. Furthermore, stoichiometric and catalytic bond forming reactions involving B–H, N–H and O–H bonds as well as carbon nucleophiles are presented.

## Abstract 2

Pnictogen pincer complexes exhibit unique electronic structures which allow activation of a range of E–H bonds and carbon nucleophiles.

## Graphical abstract

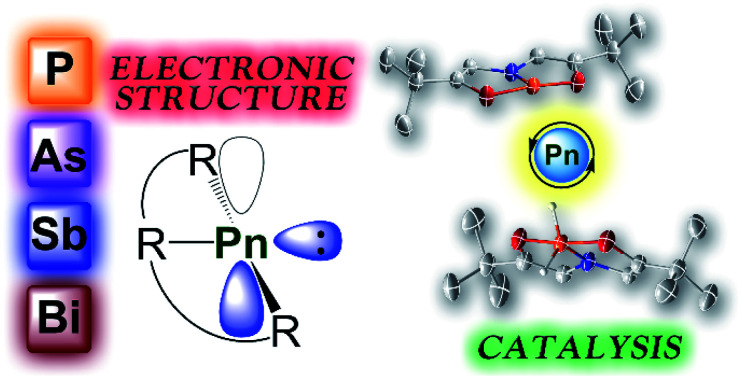

## 1. Introduction

Meridionally coordinating pincer ligands are well established in modern transition metal chemistry and have found numerous applications, *e.g.* in catalysis and small molecule activation.^1–3^ Due to their high rigidity, well-defined complexes are readily accessible, and their reactivity can be easily modified by variation of the ligand substituents.^4^ Many examples of this class of compounds are suitable for bond activation reactions due to their partially filled d-orbitals which allow for biphilic bond activation reactions due to their small HOMO/LUMO gaps and the accessibility of multiple redox states.^5^

**Scheme 1:**
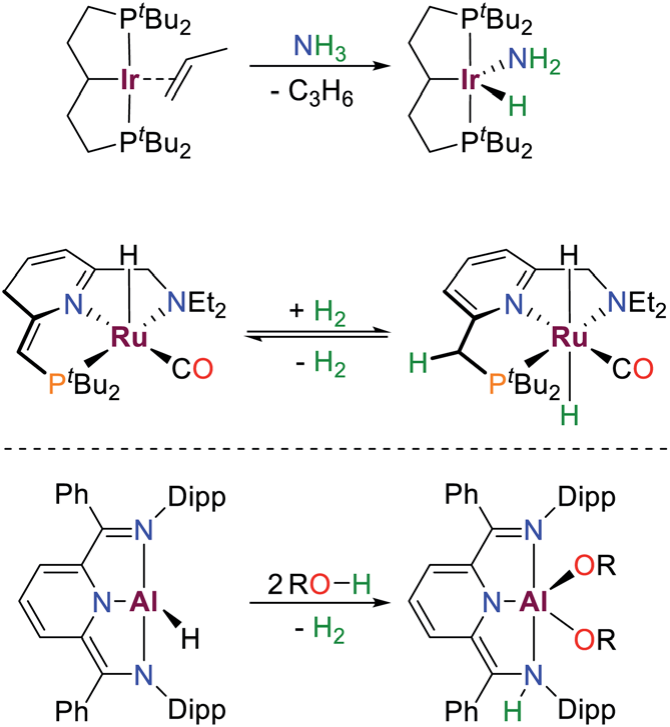
Examples of small molecule activation by transition metals and aluminium following oxidative addition (top) and cooperative pathways (middle, bottom), R = Ph, *p*-OMeBn, *p*-CF_3_Bn.^6–9^

These bond activation processes are usually accompanied by a change of the metal oxidation state, *e.g.* oxidative addition (Scheme 1).^6–9^ Besides tuning the steric profile and the redox potentials of the respective transition metal, the ligands can also be *directly* involved in bond activation or electron storage processes *via* metal–ligand cooperation, which can result in *e.g.* redox–neutral cooperative heterolytic bond scission or the addition/removal of electrons, respectively.^10^ This has allowed access to numerous highly active catalysts for diverse bond activation reactions with (base) metals in which there is a retention of the metal oxidation state, as exemplified by Milstein's seminal (de)-hydrogenation ruthenium platform (Scheme 1).^7,8,10–13^

**Scheme 2:**
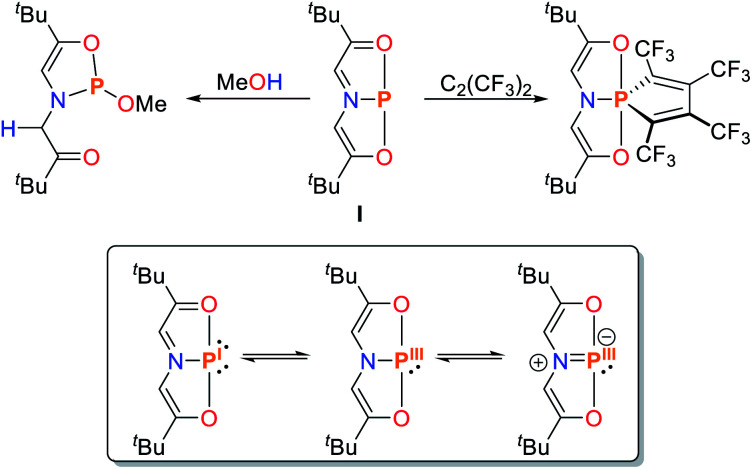
Selected reactions of the T-shaped phosphorus(i) compound **I** reported by Arduengo and the limiting resonance structures.^20,21^

Over the last decades reactivities thought to be exclusive to transition metals have also been realized for main group elements, *e.g.* cooperative bond activation and small molecule activation (Scheme 1).^9,14–19^ The utilization of pincer ligands in p-block chemistry offers the possibility to further develop bond activation reactions by main group elements, including ligand cooperativity. Pincer ligands are usually coordinated to transition metals through salt elimination reactions or acid–base chemistry. Due to the high availability of halide compounds of the main-group elements similar “coordination” reactions with pnictogens are feasible (with the exception of aromatic C–H activation). Initial reports on group 15 elements bearing geometrically constrained tridentate, meridionally coordinating pincer ligands were published by Arduengo, Baccolini, Contreras, Schmidpeter and Wolf.^20–29^ The addition of PnCl_3_ (Pn = P, As, Sb, Bi) to HN(CH_2_CH_2_C(O)R)_2_ (R = Ad, ^*t*^Bu, Ph) yielded geometrically constrained pnictogen species coordinated by an ONO pincer ligand (**I**, Pn = P, Scheme 2). While the lighter elements showed full conversion upon addition of one equivalent of ligand, the reaction with BiCl_3_ required three equivalents to yield a 9-coordinate species.^20^

These compounds are planar, *C*_2v_ symmetric species and therefore represent molecular mimics for the transition state of edge-inversion observed for trigonal pnictogens and exhibit remarkable reactivity (for R = Ad the inversion activation barrier was experimentally determined to be Δ*H*^‡^ = 23.4 ± 1.6 kcal mol^−1^).^30^ In Arduengo's landmark publication the corresponding synthetic procedures, molecular structures and reactivities were comprehensively reported. The phosphorus derivative **I** undergoes numerous intriguing reactions, including alkyne coupling and O–H bond scission of methanol (Scheme 2). With the rise of main-group metal mediated E–H bond activation in recent years this type of compound was revisited and further investigated.^16,31^

This perspective article covers the most recent developments in the field of “pincer” compounds of the group 15 elements, including a description of the effect of geometric perturbation on the electronic structure of the pnictogen complexes and the resulting orbital configurations. The rich reactivity towards E–H bonds and carbon nucleophiles is described and contextualized with theoretical results.

## 2. Effects of geometric perturbation on bonding

**Fig. 1:**
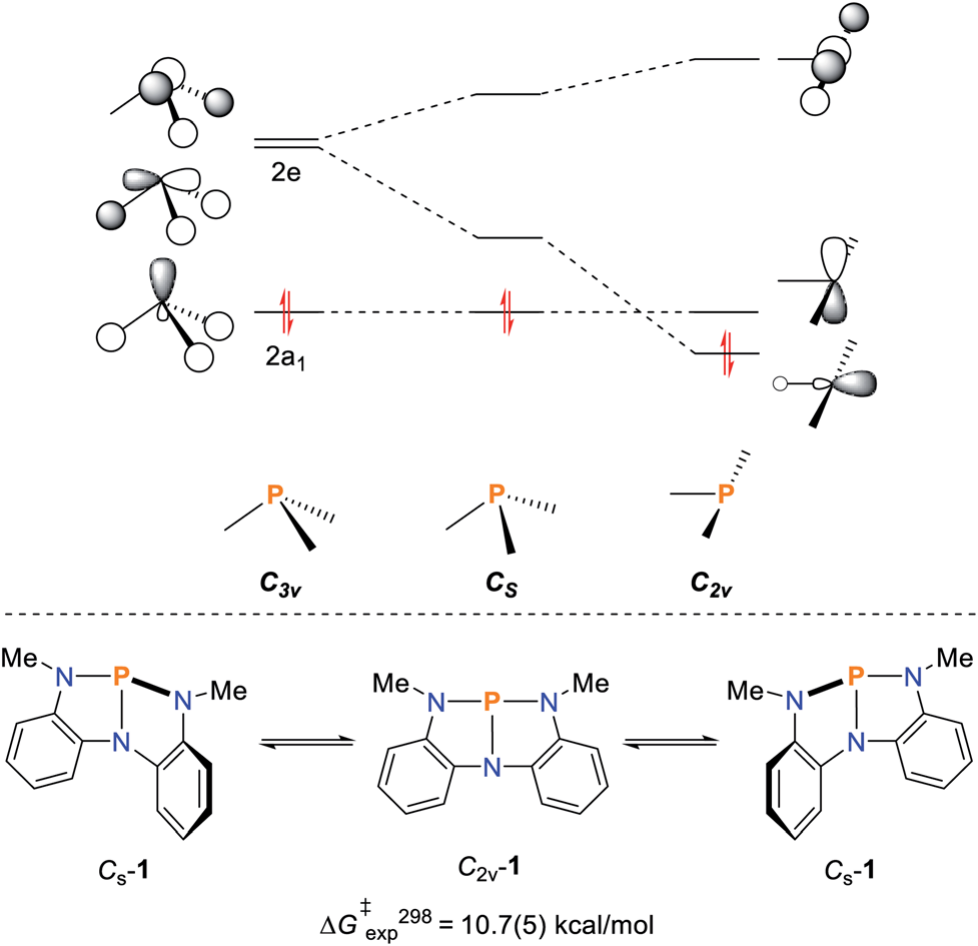
Qualitative frontier orbital diagram of a σ^3^–Pn compound and the effect of non-trigonal perturbation towards a T-shaped structure (top). Bending mode of **1** resulting in *C*_2v_-**1** (bottom).^32,36^

The rational design of geometrically constrained pnictogen platforms to facilitate the activation of small molecules requires a fundamental understanding of the electronic structure underpinning this reactivity. Utilization of tridentate, meridionally coordinating pincer ligands allows for the tuning of the bond angles around the phosphorus center which appear to be crucial for the induction of the observed reactivity (*vide infra*). In a trigonal coordination mode (*C*_3v_; characteristic of ER_3_ compounds), the lone pair of the pnictogen atom is represented by an orbital of 2*a*_1_ symmetry while the degenerate orbital set 2e consists of antibonding orbitals with respect to the pnictogen–ligand bonds (Fig. 1). The distortion towards *C*_s_-symmetry induces a lifting of degeneracy in these antibonding orbitals while the lone-pair energy is not significantly affected. Full perturbation towards a T-shaped geometry results in a considerably reduced HOMO/LUMO gap, reminiscent of the electronic configuration of transition metals,^32^ and related main-group compounds such as cyclic alkyl amino carbenes,^33,34^ which display biphilic reactivity.

The discrete ordering and energetic difference of the orbitals, however, is heavily dependent on the nature of the ligand and the pnictogen center. Upon 2^nd^ order mixing, the p-type orbital originating from the 2e set can interact with additional orbitals of π-symmetry, thereby allowing for fine tuning of the electronic structure upon pincer ligand derivatization.^35^

If the LUMO is sufficiently lowered by geometrical constraints, reduction affording a Pn(i) complex can occur, as initially proposed for **I** (electromorphism), which is generated upon addition of PCl_3_ to the diketone precursor.^20,21^ DFT computations by Sakaki show that, within the Kohn–Sham frontier orbitals, two phosphorus lone pairs can be located with respective p-type and sp-hybrid character. The P–N and P–O bonds show distinct features of coordinate bonding, yielding a suitable description of **I** as a P(i) compound.^37^ The validity of the above-mentioned orbital considerations has been demonstrated by phosphorus K-edge X-ray absorption near-edge structure (XANES) spectroscopy which is a sensitive tool to probe the electronic structure and nature of chemical bonding. Due to the excitation of an electron in the P 1s core orbital, information about the relative energetic ordering of unoccupied virtual orbitals in different compounds can be obtained.^38,39^ Since these transitions obey dipole selection rules, excitations to virtual orbitals with significant P 3p character are intense and are therefore easy to probe.^40^

Upon comparison of the pre-edge energies of P(NMePh)_3_ and **1** a significant bathochromic shift of 1.1 eV was observed for **1**. TDDFT calculation reveal that this energy shift is directly linked to the energetic lowering of one of the σ*_P–N_ orbitals due to geometric distortion towards *C*_s_ symmetry.^32^

**Fig. 2:**
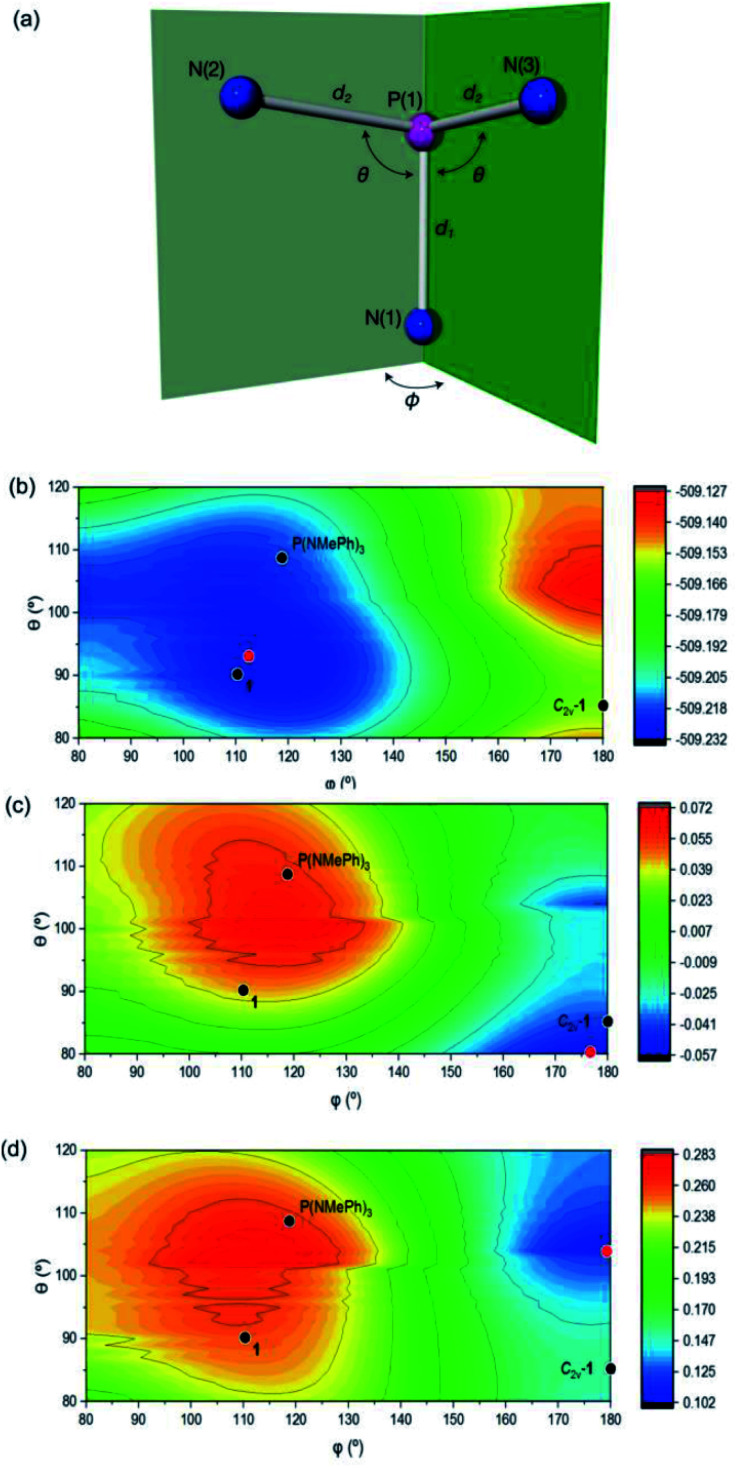
(a) Definition of geometrical parameters used to index the computational structures of P(NH_2_)_3_ within local *C*_s_ symmetry. Hydrogens are omitted for clarity. (b, c and d) Contour maps depicting the (b) total electronic energy (*E*_tot_), (c) LUMO energies (*E*_LUMO_), (d) Δ*E*_HOMO/LUMO_ for P(NH_2_)_3_ structures with *C*_s_-symmetry (*N* = 4141 discrete input structures in the range 80° ≤ *θ* ≤ 120° and 80° ≤ *ϕ* ≤ 180° at 1° increments). Energies are shown in units of Hartrees. Points corresponding to the structures of P(NMePh)_3_, **1** and *C*_2v_-**1** are superimposed as black points. The red points correspond to the absolute minimum. Used with permission from K. Lee, A. Blake, A. Tanushi, S. McCarthy, D. Kim, S. Loria, C. Donahue, K. Spielvogel, J. Keith, S. Daly and A. T. Radosevich, Validating the Biphilic Hypothesis of Nontrigonal P(iii) Compounds, *Angew. Chem. Int. Ed.*, John Wiley & Sons, 2019.

The effect of geometric perturbation on the total electronic energy, the LUMO energy and the HOMO/LUMO gap is shown by the calculated heat maps in Fig. 2 employing P(NH_2_)_3_ as a model compound. As expected, an *θ*-angle close to 90° between the ligands is preferred due to minor P-hybridization accompanied by an overall tetrahedral structure. When moving to a T-shaped geometry, the LUMO energy and the HOMO/LUMO gap gets smaller in accordance with the MO considerations shown in Fig. 1. This suggests that planarity of the molecule is most beneficial to induce biphilicity, albeit accompanied by a significant rise in electronic energy (*φ* ≤ 180, Fig. 2).

**Scheme 3:**
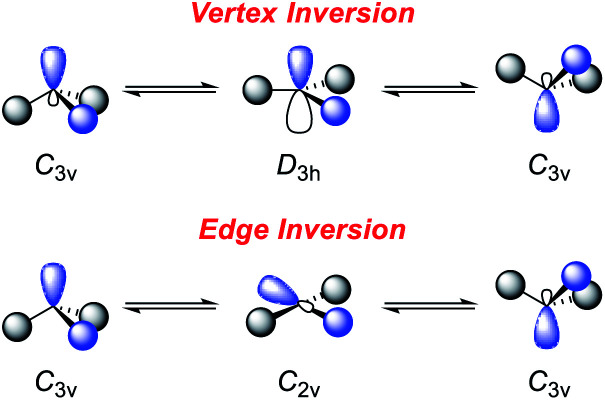
Inversion mechanisms of trigonal pnictogens.^30,47^

However, this geometry must not necessarily be provided by a rigid ligand framework, as energetically accessible bending modes, such as those observed for **1** (Fig. 1, bottom), can also induce biphilic reactivity.^36^ These molecular vibrations are commonly associated with significant activation barriers for phosphines compared to amines. Due to extensive HOMO/LUMO mixing in trigonal phosphines, perturbation of this geometry, which is stabilized by a 2^nd^ order Jahn–Teller effect, leads to high activation barriers in contrast to amines.^41,42^ The exceptionally low inversion barriers of **I** and **1** hint towards a general feature of lighter pnictogens constrained by meridionally coordinating pincer ligands. In contrast, inversion activation barriers for trigonal aryl or alkyl substituted phosphines lie in the range of 30–40 kcal mol^−1^.^43–46^ While the typical mechanism of these latter species proceed *via* a vertex inversion featuring a *D*_3h_ trigonal planar transition state, the pincer geometry enforces edge inversion, proceeding *via* a T-shaped, *C*_2v_ symmetric transition state which is usually observed for electron deficient phosphines, *e.g.* PF_3_ (Scheme 3).^30,47–49^ The angles between the phosphorus ligand bonds (*θ*, Fig. 2) cannot be easily altered by molecular vibrations. The calculated contour maps in Fig. 2 reveal that slight modifications of the geometry can greatly affect the HOMO/LUMO gap and therefore provide a guideline for further ligand design with *θ* values around 105°.^32^

**Fig. 3:**
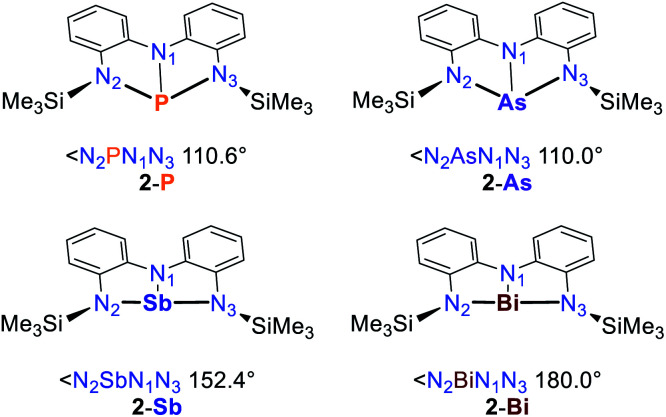
Calculated structural trends within the series of pnictogen compounds **2-Pn** ligated by an NNN pincer ligand.^50,51^

Recent studies on a series of pnictogen compounds ligated by an NNN aryl-based pincer ligand reveal interesting structural trends when moving from phosphorus to bismuth (Fig. 3).^50,51^ Since the NNN scaffold does not feature a tethering unit or very bulky substituents on the ligand backbone which enforce planarity, the nature of the pnictogen center dictates the observed geometry. For monomeric **2-P** and **2-As**, trigonal pyramidal structures are observed in the solid state. Both compounds feature a planar N_1_CCN_2_Pn ring while the remaining N_1_CCN_3_Pn is significantly twisted suggesting a possible description as masked pnictenium cations bound to a pendant amide. **2-Sb** has been shown to dimerise at low temperatures in solution and crystallizes as a dimer.

The monomeric species **2-Sb** is calculated to be essentially planar, like the bismuth analogue **2-Bi** which remains monomeric at low temperatures and in the solid state. The activation barriers for edge inversion are found to gradually decrease when moving to the heavier atoms, with the *C*_2v_ structure of **2-Bi** being slightly exergonic. This is being explained upon partitioning of energetic factors by an energy decomposition analysis (EDA).^52^ The orbital stabilization of the 3-center, 4-electron N–Pn–N bond increases when moving to heavier analogues due to a rise in Pn–E_Ligand_ electronegativity differences and the increasing s-character of the pnictogen lone-pair for heavier elements (inert pair effect).^53^ This is accompanied by a reduction in Pauli repulsion in the T-shaped geometry. In contrast, lighter elements are more reluctant to adopt a planar geometry due to increased electrostatic interactions. This leads to the observed structural trends and directly affects the reactivity due to the alterations of the LUMO energy as depicted in Fig. 1, resulting in an increased Lewis-acidity for the heavier pnictogen centers and/or a propensity for heavier pnictogens to adopt Pn(i) character when being ligated by redox-active ligands. This “redox confusion” is demonstrated by the electromorphic synthesis of the Bi(i) compound **2-Bi** from BiCl_3_ and the protonated pincer which shows coordination towards W(CO)_5_*via* a filled p-orbital but also exhibits Lewis-acidity upon binding of two equivalents of pyridine-N-oxide.^50,51^

**Fig. 4:**
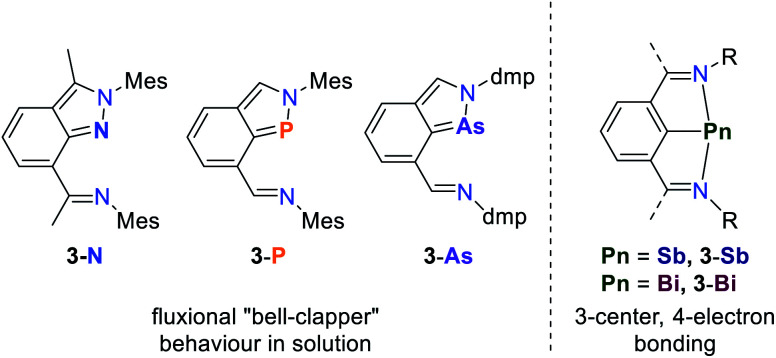
Series of geometrically constrained NCN ligated pnictogen compounds **3-Pn** and their fluxionality in solution dependent on the central element, R = ^*t*^Bu, Mes, dmp; Mes = 2,4,6-Me_3_–C_6_H_2_, dmp = 2,6-Me_2_C_6_H_3_.^54–57^

The reduced tendency of lighter pnictogens to form planar, low valent structures exhibiting 3-center, 4-electron bonding is further demonstrated for a series of NCN ligated geometrically constrained compounds (Fig. 4). While **3-N**, **3-P** and **3-As** exhibit fluxional “bell-clapper” isomerism in solution, **3-Sb** and **3-Bi** show no dynamic behavior and engage in hypervalent bonding patterns.^54–57^

### 2.1 Unprecedented redox transformations

**Scheme 4:**
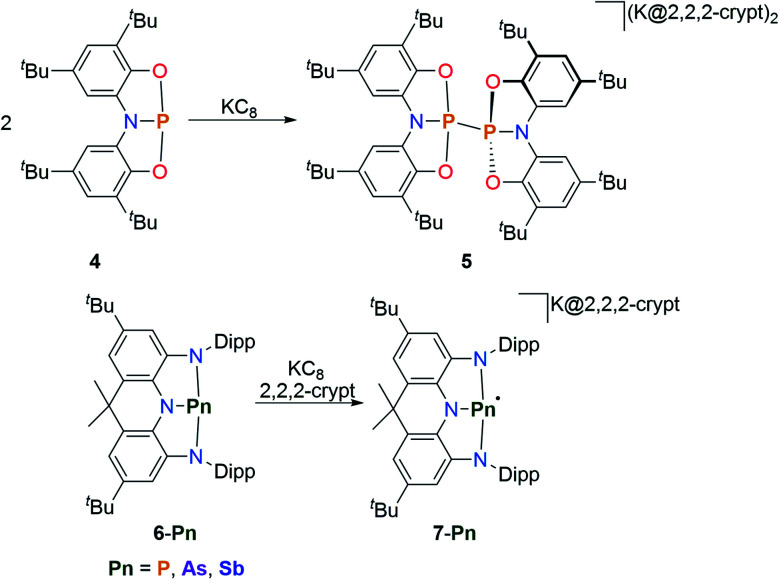
Reduction of T-shaped pnictogens species towards dimeric dianions and monomeric radical anions, 2,2,2-crypt = N[CH_2_CH_2_OCH_2_CH_2_OCH_2_CH_2_]_3_N.^60,61^

The constitution of the frontier molecular orbitals of geometrically constrained pnictogen species offers the possibility to pursue new redox transformations. The oxidation of geometrically constrained pnictogen species towards the trigonal radical cations is unprecedented^58,59^ while the synthesis of pentacoordinate derivatives upon oxidation is well established. A comprehensive coverage of the latter is beyond the scope of this article and we therefore recommend Uhl's recent review article for further reading.^31^ Since the LUMO in traditional trigonal pnictogen species are antibonding with respect to the ligands, one-electron reduction usually results in fragmentation of the PnR_3_ species. Due to the unique electronic configuration of non-trigonal pnictogen pincer compounds, which possess a low-lying empty p-type orbital at the pnictogen center, radical anions could however be generated. Initial studies on the possibility of forming reduced species were conducted by Goicoechea and co-workers.^60^ Upon reduction of **4** at room temperature, a dianionic species, **5**, is obtained upon radical coupling, without a detectable paramagnetic intermediate in solution (Scheme 4). Wang and co-workers reported on a pnictogen series ligated by a sterically demanding acridine-derived NNN pincer ligand (Scheme 4).^61^ The T-shaped compounds (**6-Pn**) feature reversible redox events in the cyclovoltammograms ranging from *E*_1/2_ = −2.28 V to *E*_1/2_ = −1.90 V, with the phosphorus analogue featuring the lowest potential. Upon reduction with potassium the corresponding radical anions are obtained which do not show any signs of decomposition in solution or in the solid state. In accord with DFT computations of the (**7-Pn**) series, the reduction takes places at the respective pnictogen center.

This is further corroborated by the recorded EPR spectra which display distinct hyperfine interactions with the pnictogen centres. The large z-component of the measured hyperfine interaction tensor of **7-P** compared to the other orientations (*a*_*z*_(^31^P) = 181 G; *a*_*y*_(^31^P) = 15.0 G) suggests a localization in a phosphorus p-type orbital. DFT calculations further show that the unpaired electrons for all studied compounds mainly reside on the pnictogen atom with contributions from the π-symmetric orbitals of the NNN pincer scaffold. The arsenic and antimony derivatives proved to be efficient reductants to obtain the unprecedented sulphur anion S_10_^2−^ upon reduction of elemental sulfur. A recent report proposes the possible isolation of a formal P(0) species upon reduction of a pyridyl-diime ligated phosphorus complex which exhibits a reversible reduction in the cyclovoltammogram.^62^

### 2.2 Implication for transition metal chemistry

Constrained pnictogen compounds have also been investigated in terms of their ability to act as ligands to transition metals. As mentioned above **2-Sb/Bi** and **3-Sb/Bi** adopt a planar geometry due to a combination of orbital and electrostatic effects. DFT suggests a significant Pn(i) character due to ligand oxidation, as initially examined by Dostál.^50,56,57,63^ Recent reports of the coordination chemistry of such Pn(i) species to transition metal carbonyl species indicate that the occupied p-orbital at the pnictogen center is capable of acting as a 2e^−^ donor ligand due to its observed metal coordination geometry, perpendicular to the molecular-plane.^21,51,57,63–68^ A recent review covers this topic comprehensively for phosphorus-based compounds,^31^ however we would like to address new reports which make use of this unusual electronic configuration beyond traditional L-type ligand coordination. **1** was derivatized by 2-pyridyl substituents to provide a confined pocket for transition metal complexation. XANES analysis of the ruthenium(ii) dichloride complex indicates the preservation of a low-lying p-type orbital at the phosphorus center upon metal complexation.^32^

**Scheme 5:**
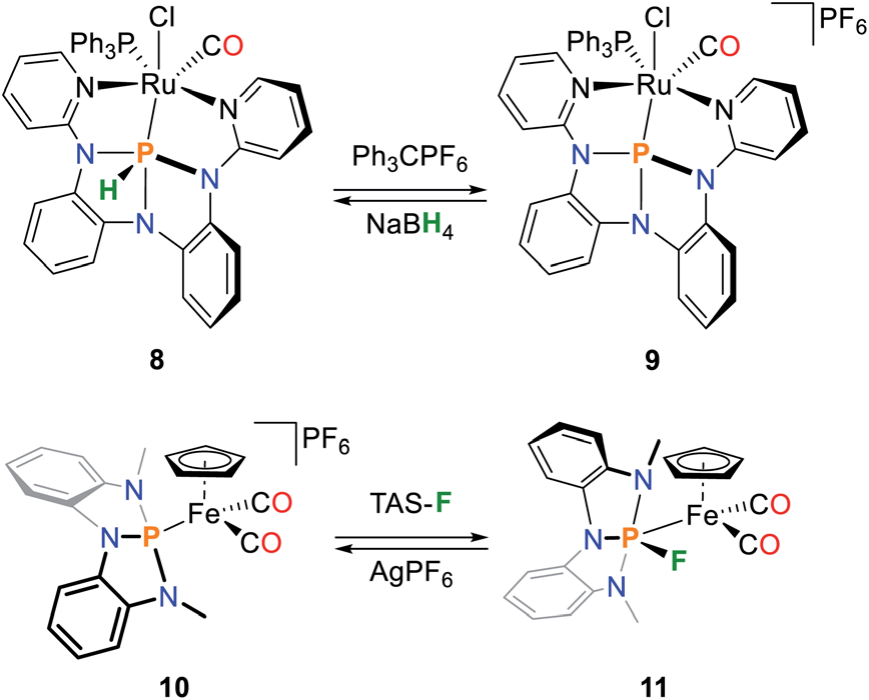
Reversible P-centred hydride and fluoride addition to transition metal complexes featuring geometrically constrained phosphorus ligands.^69,70^

Therefore, increased π-interactions with transition metal centers as well as ambiphilic reactivity patterns are expected to be accessible. The arising biphilicity is shown upon complexation of the hydride complex [RuHCl(CO)(PPh_3_)_3_].^69^ Instead of coordination of the phosphorus ligand, the insertion product **8** was obtained (Scheme 5). Based on NMR measurements and DFT calculations, the reaction product is best described as an unprecedented metallohydrophosphorane complex. The hydricity of **8** allowed for clean abstraction of the hydride ligand to give **9**, which in turn could be converted back towards **8** upon addition of an H^–^ source. A DFT analysis of the Fp complex **10** (Fp = Fe(C_5_H_5_)(CO)_2_) shows that the empty p-type orbital at the phosphorus center results in significant π* back-bonding compared to the P(NMe_2_)_3_ ligated Fp-analogue, a property which is usually encountered in electron deficient phosphines which feature low lying σ*_P–C_-antibonding orbitals.^70–73^ An L/X type ligand transformation is observed upon addition of fluoride anions, resulting in the formation of the fluoro–metallophosporane complex **11** accompanied a significant highfield shift of the phosphorus ^31^P NMR resonance by 130 ppm (Scheme 5). This results in a cancelling of π-back donation from the metal center as shown by DFT studies. Upon addition of silver cations, **11** is converted back into **10**.

## 3. Stoichiometric and catalytic E–H bond activation

The activation mechanism of E–H bonds by geometrically constrained pnictogen compounds is highly dependent on the respective HOMO and LUMO energy levels and the polarity of the element–hydrogen bond in question.

**Scheme 6:**
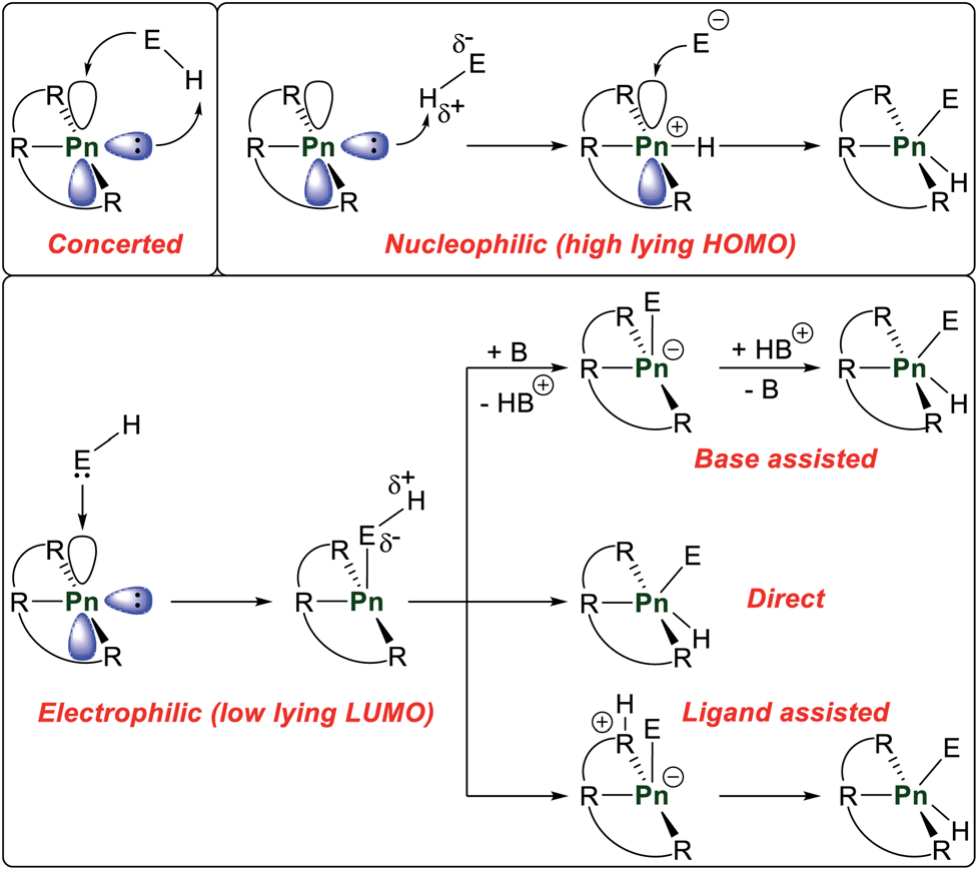
Possible activation of E–H bonds by geometrically constrained pnictogen pincer compounds.

Three different activation pathways of the substrate are possible and are subsequently discussed for phosphorus compounds that activate protic E–H bonds in which phosphorane reaction products represent the thermodynamic minimum on the potential energy surface (Scheme 6). A concerted mechanism in which the occupied sp-hybrid orbital and the empty p-orbital overlap with the antibonding E–H σ*- and bonding E–H σ-orbital, respectively, to directly yield a P(v) compound can be envisioned, mimicking oxidative addition reaction in transition metal complexes. However, this reaction mechanism is expected to necessitate very small HOMO/LUMO gaps to be operative. If the sp-hybrid orbital exhibits favourable basicity a nucleophilic attack *via* deprotonation of the substrate is possible to generate a cationic tetragonal intermediate which is followed up by capture of the generated anion. The last possible mechanism consists of initial electrophilic attack of the substrate *via* the empty phosphorus p-orbital which further polarizes the E–H bond and might result in isomerization towards the phosphorane (direct). Other possible scenarios are that the protic hydrogen atom is either abstracted by an external base (base assisted), *e.g.* an amine, or a basic pincer functionality (ligand assisted). Upon proton shuffling the final P(v) compound can be accessed.

### 3.1 Activation of B–H bonds

Radosevich and co-workers showed that **I** reacts with ammonia borane (AB) to yield the formal oxidative addition product **12** with no intermediates to be detected when the reaction is followed by NMR spectroscopy.^74^

**Scheme 7:**
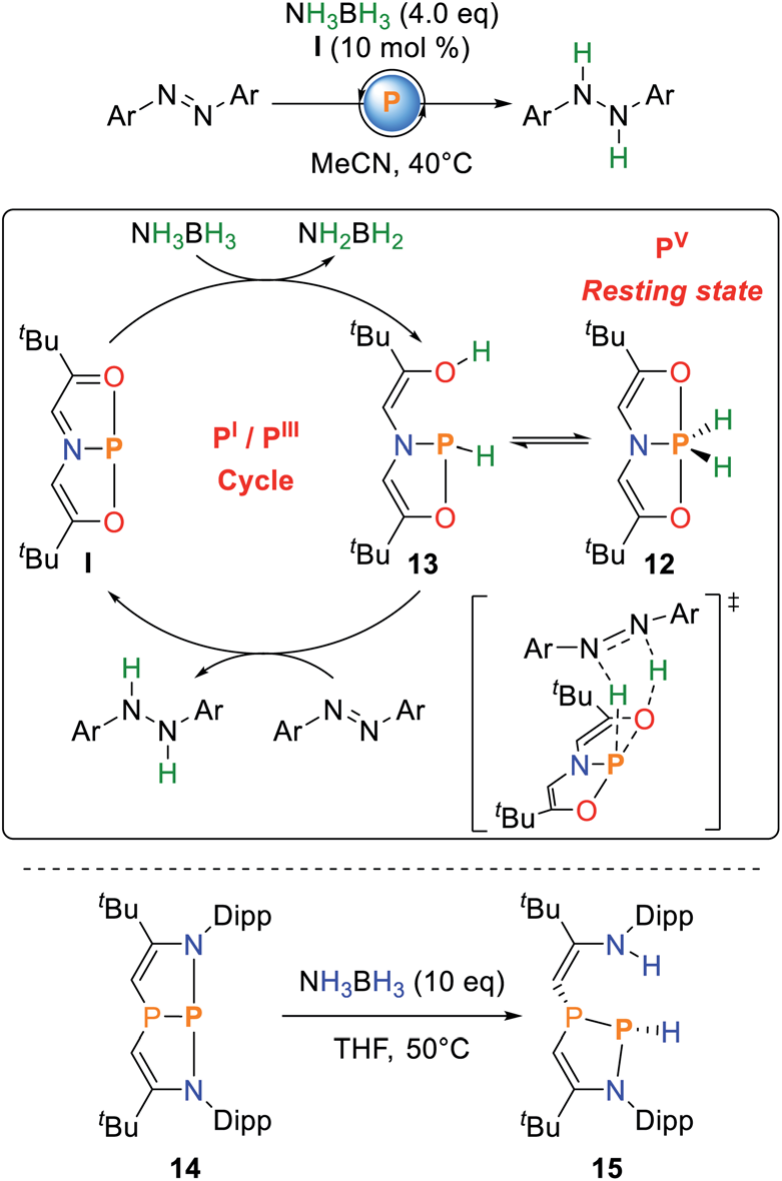
Catalytic hydrogenation of azobenzene from ammonia–borane mediated by **I** (top) and cooperative addition to the diazadiphosphapentalene **14** (bottom).^74,75^

Upon exposure to azobenzene, selective hydrogenation towards diphenylhydrazine is observed (Scheme 7). The reaction proceeds at mild conditions but with a relatively high catalyst loading of 10 mol%. Monitoring the catalytic process *via* NMR spectroscopy showed the clean disappearance of the ^31^P NMR resonance of **I** and selective formation of **12**, suggesting that the P(v) dihydridophosphorane is the resting state of the catalytic cycle. Consequently, **12** could also be used as a catalyst, albeit with reduced efficiency. A comprehensive study of the reaction mechanism was computationally performed by Sakaki and co-workers.^76^ It was shown that AB dehydrogenation does not occur solely at the phosphorus center but rather is accomplished upon a P–O cooperative dehydrogenation of AB to yield the phosphine **13**. The dihydridophosphorane **12** represents an off-cycle resting state of the catalyst and does not allow for hydrogenation *via* a five membered transition state due to a computed high activation barrier. Consequently, **12** needs to be converted back to **13***via* AB assisted isomerization which explains the lower catalytic efficiency when **12** is used instead of **I**.^37,74^

The diazaphosphapentalene **14** reported by Kinjo follows a related activation pathway for AB dehydrogenation. Upon addition of AB to **14**, the cooperative addition along the P–N bond is observed to give **15** (Scheme 7). Unlike **12**, **15** is thermally unstable and degrades to a mixture of products, even at low temperatures, possibly induced by imine-tautomerization also observed for N–H activation processes (*vide infra*).^75^

**Scheme 8:**
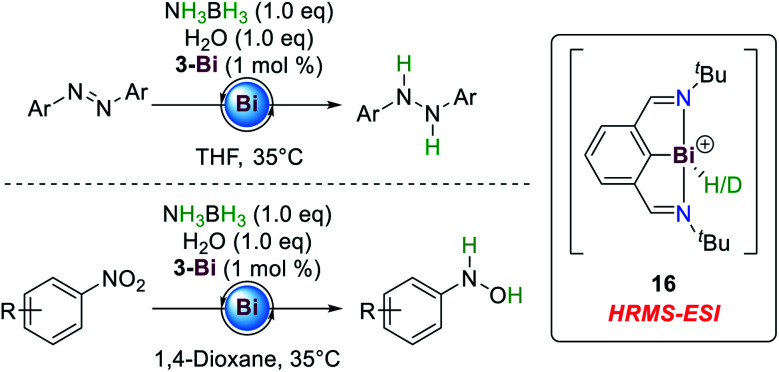
Hydrogenation of azoarenes and nitroarenes by ammonia-borane employing a Bi(i/iii) redox system.^77^

Another Pn(i/iii) catalytic cycle was described by Cornella and co-workers, who showed that compound **3-Bi**, initially reported by Dostál and co-workers,^56^ is also an active catalyst for the hydrogenation of azoarenes and nitroarenes from AB towards hydrazines and *N*-arylhydroxylamines, respectively (Scheme 8).^77^ While Bi(iii) hydrides usually favour the extrusion of dihydrogen to yield the corresponding (dimeric) Bi(i) compounds,^56,78^ reductive elimination proves to be sufficiently slow at **3-Bi** to allow for hydrogenation reactions.

The reactions proceed at low catalyst loadings (1 mol%) and temperatures. The stoichiometric addition of water to the reaction mixture results in significant acceleration of the reaction times, probably owing to beneficial hydrogen bonding between AB and water during the catalytic cycle. In contrast to conventional transition metal complexes, this catalytic protocol tolerates weak bonds such as carbon iodide functionalities and halogenated solvents. Also, the selective formation of *N*-arylhydroxylamines is rather uncommon in transition metal catalyzed transformations.^79^ Further investigation by kinetic isotope effect and crossover experiments suggest that the substrate is not involved in the rate determining step and rather water, AB and **3-Bi** dictate the overall rate law of the reaction. A monomeric Bi(iii) dihydride complex was proposed to be a catalytically active intermediate. However, under both stoichiometric and catalytic conditions such a species could not be observed. A cationic monohydride and its corresponding deuteride **16** could be detected by HRMS-ESI spectrometry during catalysis.

**Scheme 9:**
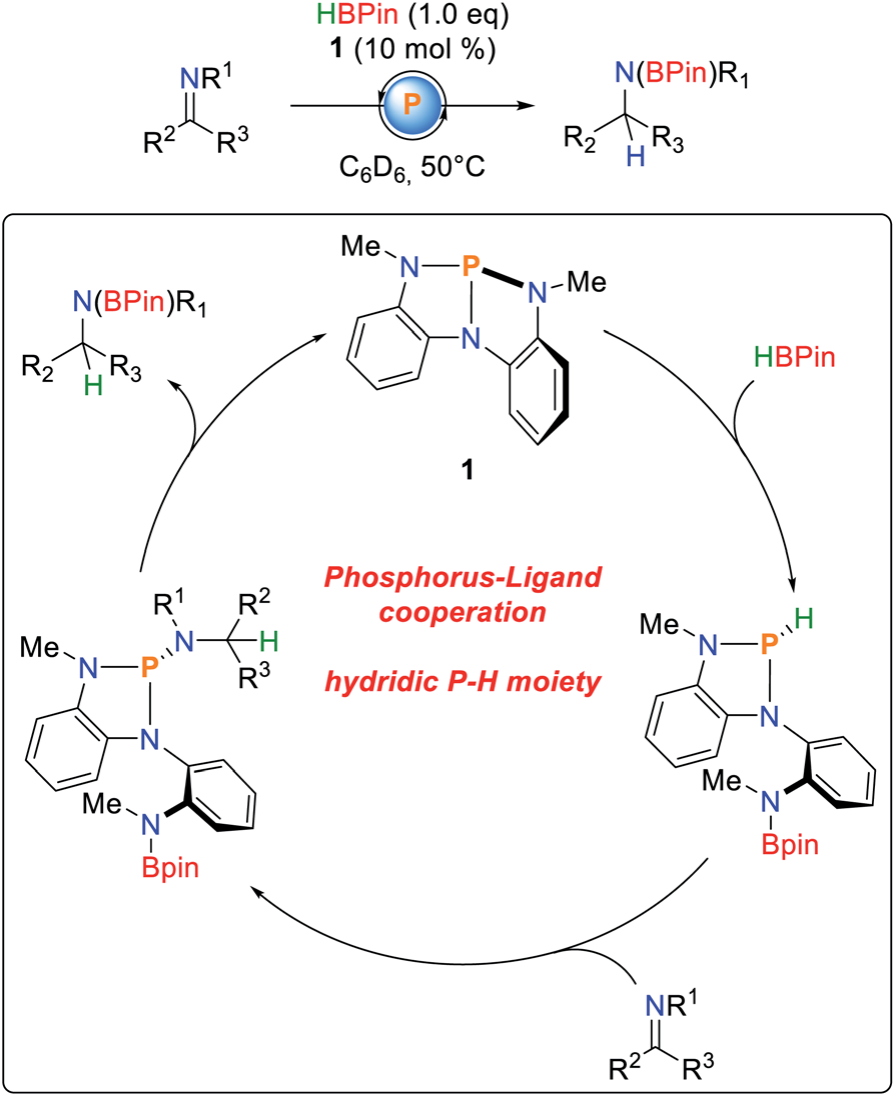
Catalytic hydroboration of imines by **1**.^80^

Besides the activation of protic E–H bonds, it was demonstrated that **1** is also able to activate bonds of inverse polarity, namely boranes (Scheme 9).^80^ As observed for O–H and N–H bond activations (*vide infra*), the B–H bond adds along the P–N bond of **1**, obeying a linear dependence of the reaction order on the concentration of the starting materials. The hydricity of the phosphine could be shown by NBO calculations,^81^ as well as successful hydrodechlorination of chloroform after HBPin addition. Furthermore, imines cleanly insert into the P–H bonds to give the corresponding triazaphospholenes which, depending on the substrate, exhibit limited stability and cleanly regenerate **1** and the hydroboration product. Kinetic analysis suggests a first order rate law due to cooperative elimination.

### 3.2 Activation of N–H bonds

The ONO pincer ligated phosphorus compound **I** activates ammonia and amines to give the corresponding P(v) amide hydride compounds.^82^ The amide substituents are coordinated in the equatorial plane in all cases and engage in orbital stabilization *via* N_π_–σ*_N–P_ interaction as shown by NBO analysis. The thermodynamic driving force of N–H bond scission was probed by isothermal titration calorimetry of **I** with *n*-propylamine. The reaction was found to be strongly exothermic with Δ*H* = −10.6 ± 0.1 kcal mol^−1^ due to the favourable formation of phosphorus(v) stabilized by the highly electronegative oxygen atoms. The ammonia derived compound **17** cannot be converted back to the starting material, and sublimes at elevated temperatures.

When BnND_2_ is used as an amine source no incorporation of deuterium is observed in the olefinic backbone, thereby ruling out the possibility of direct ligand participation in the bond activation akin to Milstein's ruthenium complex.^7,8^ The reaction follows a first order rate law in **I**. Surprisingly, the overall rate law is dependent on the cubic concentration of the amine and features a large transition state entropy (Δ*S*^‡^ = −72 ± 2 cal mol^−1^ K^−1^).

**Scheme 10:**
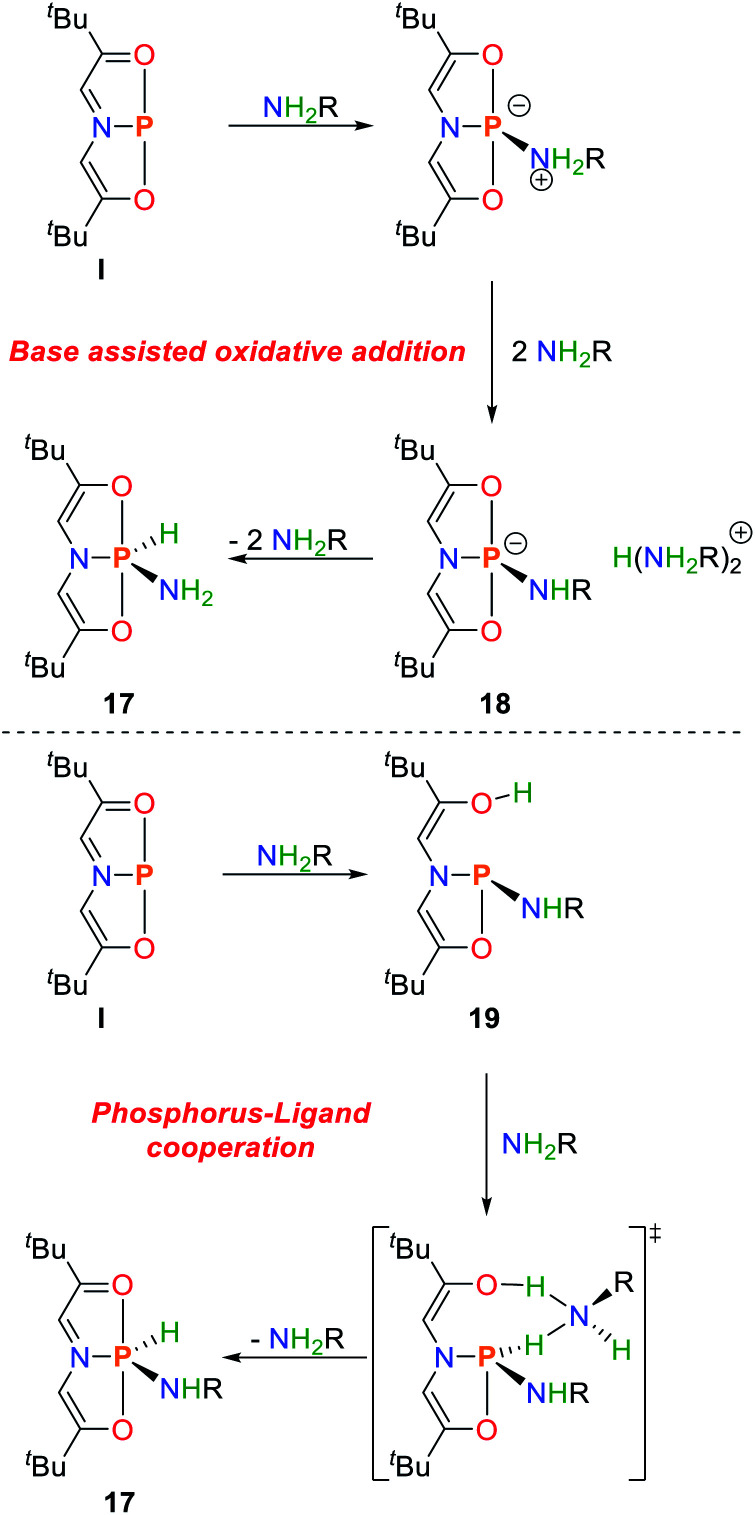
Proposed mechanisms of oxidative addition of amines by **I**.^82,83^

The mechanism was further investigated by DFT calculations and shows that the initial coordination *via* the nitrogen atom towards the phosphorus center is favoured over amine deprotonation, representing an electrophilic activation of NRH_2_ by the available p-type vacant orbital of the phosphorus platform. The third order in amine was rationalized by a base assisted deprotonation of the coordinated amine by excess base to give **18** which allows for proton shuttle and generation of the final compound **17** (Scheme 10).

Another mechanistic proposal derived by DFT calculations favours a cooperative activation of the nitrogen–hydrogen bond along the P–O bond resulting in bond scission with retention of the phosphorus oxidation state, generating **19**. Subsequent base assisted proton shuttle gives **17**. This mechanism was found to be associated with lower barriers, however a high dependence on amine concentration and solvent appears to dictate the operative mechanism (Scheme 10).^83^

**Scheme 11:**
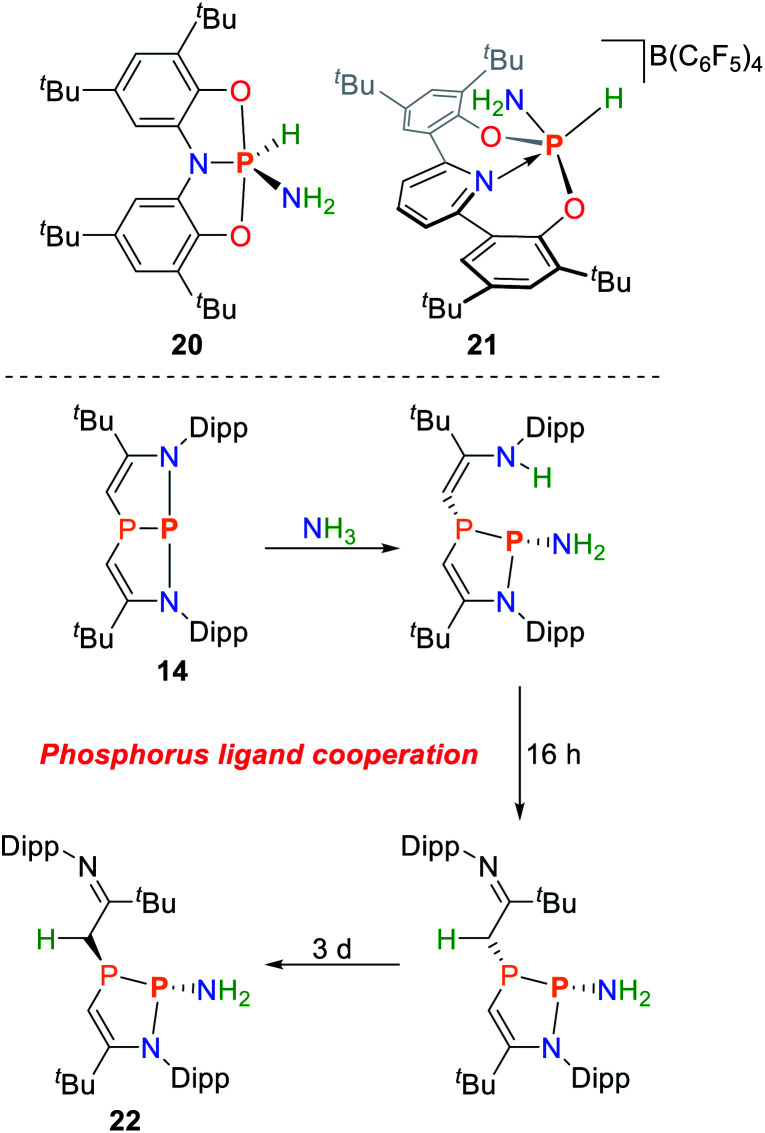
Oxidative addition of ammonia towards ONO pincer platforms (top) and cooperative activation by a diazadiphosphapentalene (bottom).^84,86,87^

Besides Arduengo's ONO pincer system **I**, an aromatic ONO pincer ligand shows to also be capable of ammonia activation to give a P(v) amide hydride species **20** (Scheme 11).^84^ Exposure towards dynamic vacuum at 100 °C restores the starting material in small amounts. The reaction enthalpy of the reaction was calculated by DFT to be Δ*H* = −22 kcal mol^−1^.^85^ The analogous reaction with the heavier arsenic analogue is endothermic by Δ*H* = +21 kcal mol^−1^. This can be attributed to the subsequent stabilization of the s-orbital when moving to heavier elements (inert pair effect) and the significantly reduced bond strengths of As–N and As–H bonds when compared to phosphorus.

The concept of inducing biphilicity upon introducing geometric constraints has also been extended towards phosphenium cations, accessible upon halide abstraction from the corresponding chloro-phosphines. Upon exposure to ammonia the phosphorus(v) amide hydride **21** is obtained (Scheme 11). Interestingly, this reaction could be reversed upon heating to 70 °C.^86^

The diazadiphosphapentalene derived NPN pincer ligated phosphorus(iii) center, **14**, showed to be capable of activating ammonia at room temperature.^87^ In contrast to the structurally related ONO system **I**, the reaction rate depends linearly on the concentration of both reagents. The initial species that is formed represents a cooperative ammonia activation product produced along the P–N bond, which could also be characterized crystallographically. The activation parameters of the cooperative addition were measured to be Δ*G*^‡^_298_ = 22.8 ± 1.8 kcal mol^−1^, Δ*H*^‡^ = 13.8 ± 0.9 kcal mol^−1^ accompanied by a significantly smaller entropic contribution when compared to the ammonia activation by **I** (Δ*S*^‡^ = −30.3 ± 3.1 e.u.). The reaction mechanism as determined by DFT calculations confirms the proposed cooperative bond activation pathway (Scheme 11). Continued stirring results in enamine–imine tautomerization followed by a steric rearrangement giving the phosphoramide **22** which represents the global minimum on the potential energy surface. Both isomerization reactions are associated with significantly higher activation barriers, resulting in reaction times of up to several days.

**Scheme 12:**
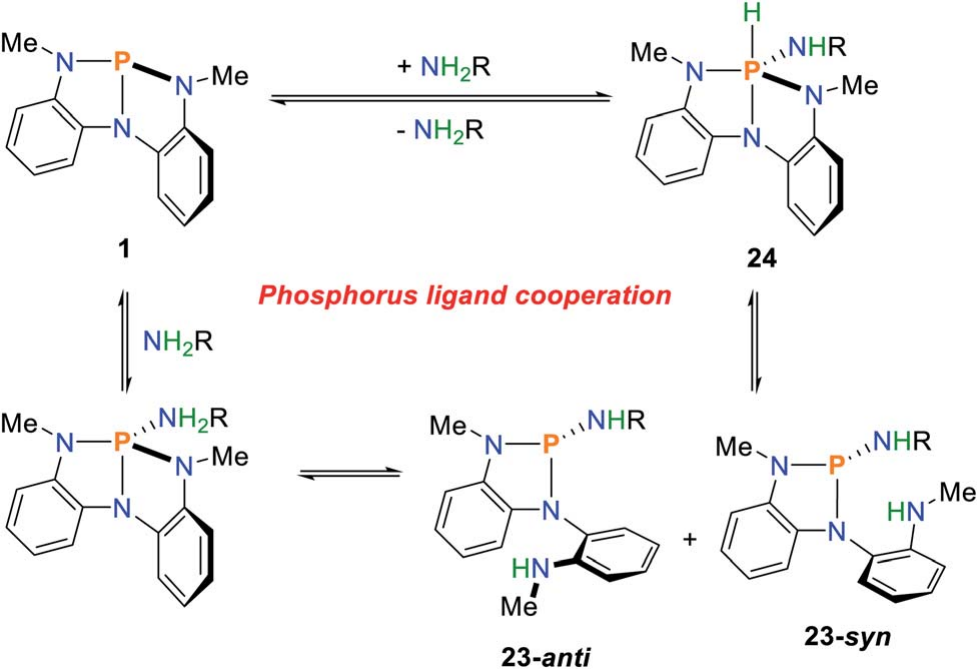
Cooperative N–H bond activation of primary amines mediated by **1**, R = H, alkyl, aryl.^36^

A similar reaction sequence was found for the phosphorus compound **1** (Scheme 12).^36^ Upon addition of primary amines, a mixture of the two cooperative addition products, **23-syn** and **23-anti** is generated, with the *anti*-product being favoured due to steric interactions. In the solid state, the phosphorane product **24** can be characterized which represents the thermodynamic minimum of the observed reaction products. Upon dissolving of crystalline **24**, a slow conversion towards the mixture of cooperative addition products **23-syn** and **23-anti** can be observed by NMR spectroscopy. The addition of different amines leads to slow exchange of the amide substituent over the course of several days.

A Hammett-analysis of the exchange reactions reveals that electron-rich amines bind more favourable to the phosphorus compound. The overall reversibility of this reaction was shown upon heating of the *tert*-butyl amine activation product in toluene under a dynamic nitrogen flow which afforded the starting material **1** in quantitative yields. The activation of ammonia can be observed in solution by ^31^P and ^1^H NMR spectroscopy, however isolation of pure material is hampered by the presence of another species which was proposed to be an NH bridged condensation product.

### 3.3 Activation of O–H bonds

**Scheme 13:**
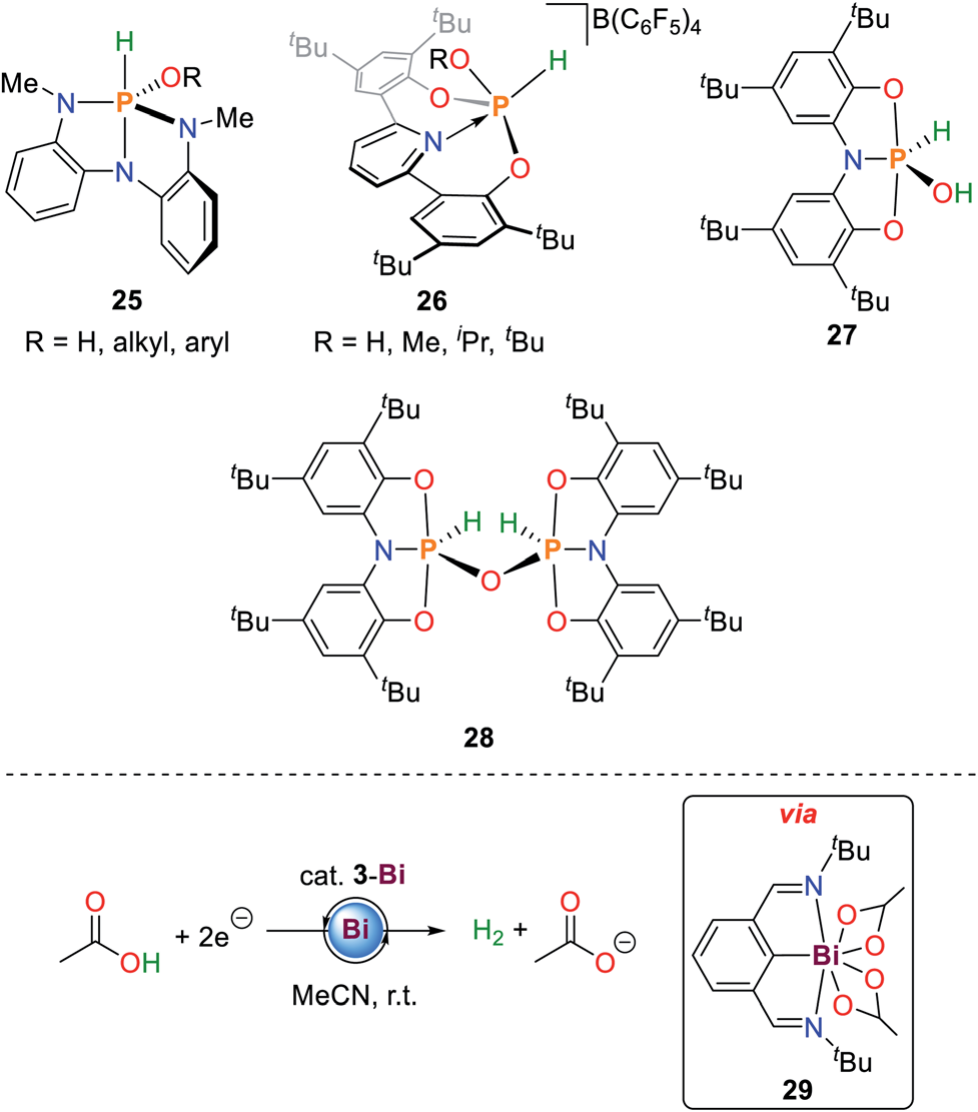
Reaction products of oxidative addition of water and alcohols to geometrically constrained phosphorus compounds (top) and electrocatalytic generation of dihydrogen from acetic acid by **3-Bi**.^36,84,86,89^

In addition to N–H bond activation, **1** is also able to activate alkyl and aryl alcohols (**25**, Scheme 13).^36^ The mechanistic details resemble those observed for primary amines described above. The measured equilibrium constants between *syn*- and *anti*-activation products of HO^*t*^Bu reveal a distinct preference for the sterically less demanding reaction product (Δ*H* = 4.5 ± 1 kcal mol^−1^ and Δ*S* = 13.8 ± 5 cal mol^−1^ K^−1^).

Since the initial report of decomposition of **I** towards phosphoric acid when exposed to water, only few platforms have been reported that are capable of water O–H bond activation.^88^

In analogy to ammonia activation, geometrically constrained phosphenium cations proved to be capable of activating O–H bonds. Besides water, aliphatic alcohols could be cleanly activated to give the corresponding phosphorus(v) alkoxide hydride species **26** (Scheme 13).^86^

The hydroxide hydride phosphorane **27** is obtained upon exposure of the geometrically constrained phosphine to one equivalent of water.^84^ In contrast to the ammonia activation product **20**, addition of another equivalent of starting material and heating to 70 °C results in the formation of the oxygen bridged dimeric P(v) compound **28** (Scheme 13). This product is also obtained upon heating **20** under dynamic vacuum at 100 °C over 36 h. The reaction can be reversed upon addition of stoichiometric amounts of water.

**3-Bi** is a rare main group element-based hydrogen evolving electrocatalyst for the reduction of acetic acid towards dihydrogen and acetate.^89–91^ Upon double O–H bond scission of acetic acid the 7-coordinate Bi(iii) species **29** is formed accompanied by the release of dihydrogen which can be reduced by two electrons to regenerate **3-Bi** (Scheme 13).

## 4. Pn–C bond formation reactions

The recent reports on geometrically constrained pnictogen compounds mainly focus on the activation of polar E–H bonds (E = B, N, O) to enable reaction patterns related to established transition metal chemistry. However, the reaction of such species with carbon-based reagents have received comparably less attention since Arduengo's initial studies on constrained pnictogens ligated by an ONO ligand.^20,21^

As shown above, **I** is able to dimerize electron-deficient alkynes upon phosphole formation, hinting towards a versatile reactivity of geometrically constrained pnictogen species.^21^

**Scheme 14:**
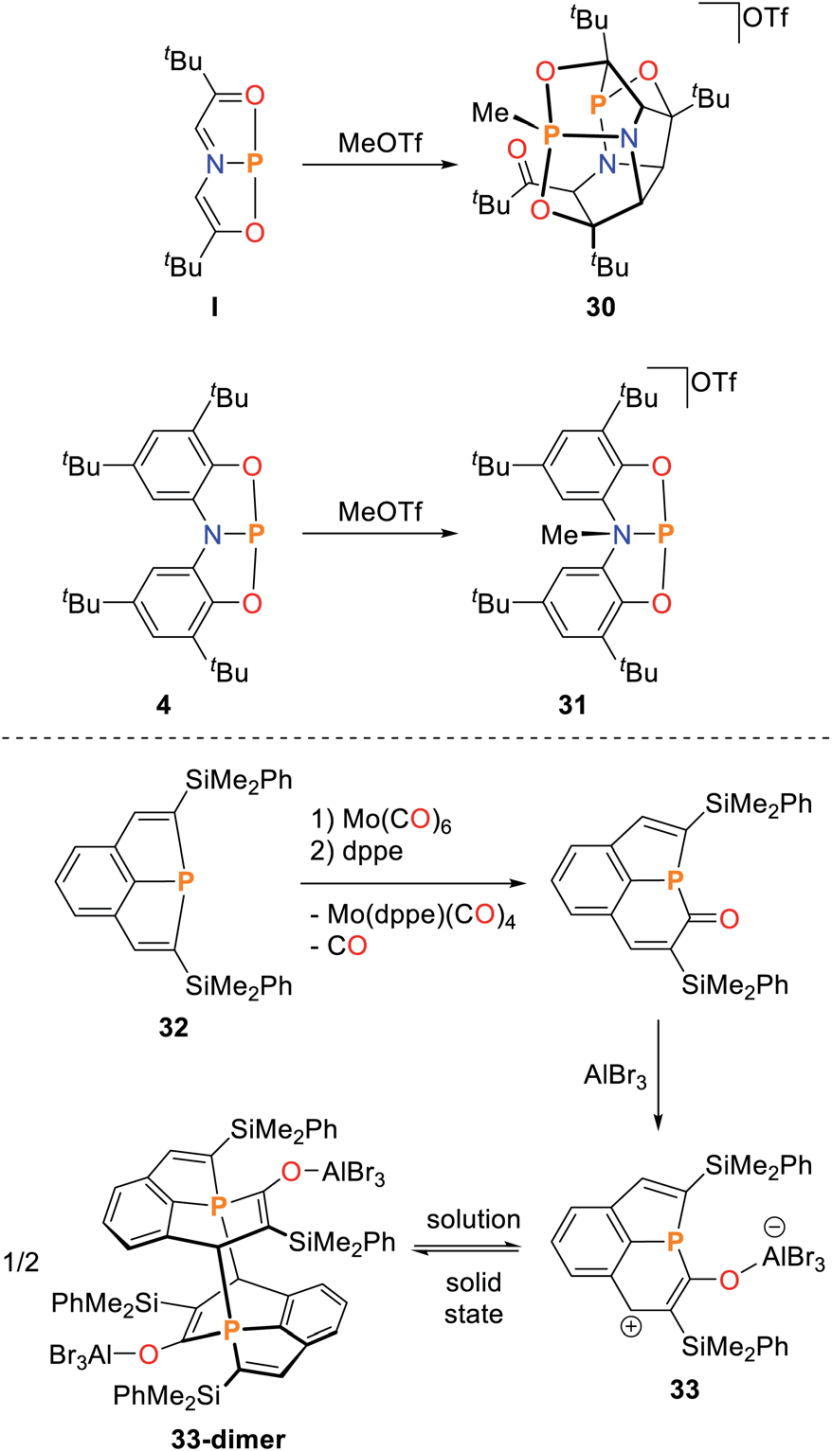
Reactions of geometrically constrained pnictogen species towards carbon-based nucleohpiles, R = Me, ^*t*^Bu, Ph, dppe = diphenylphosphinoethane.^85,92,93^

**Scheme 15:**
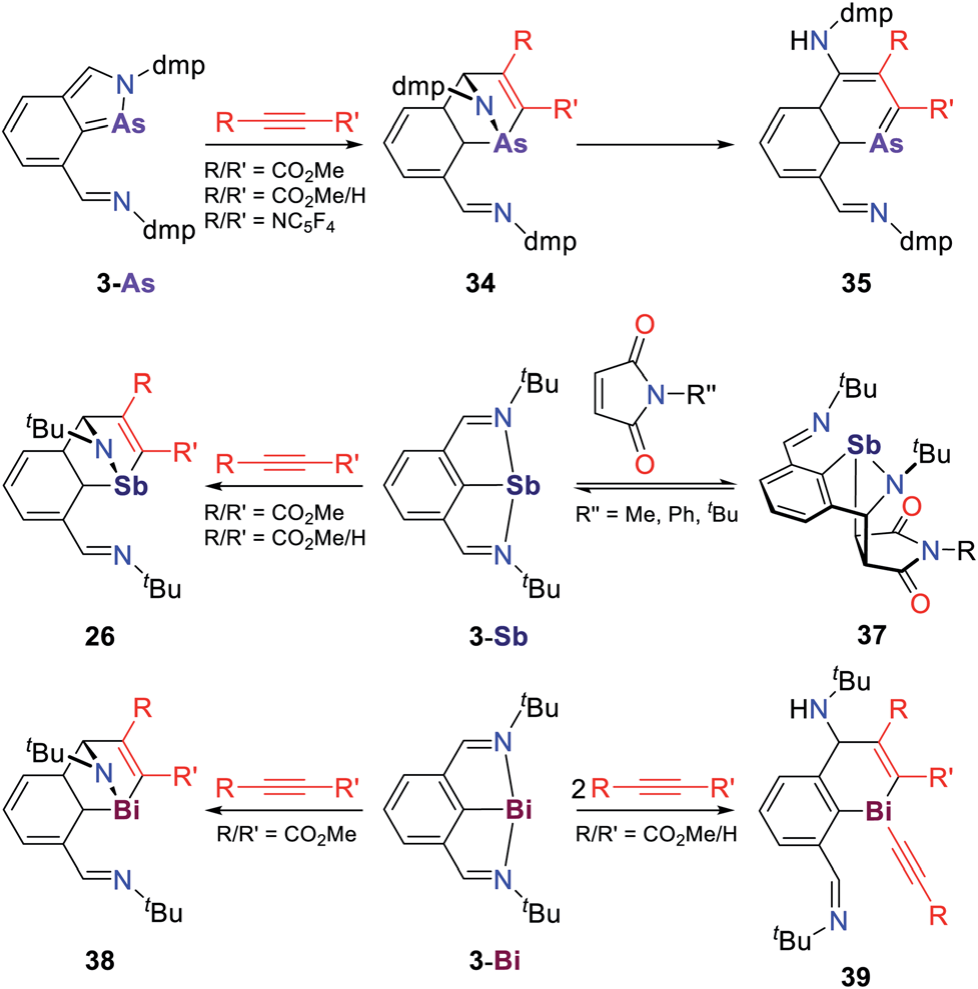
Reaction of NCN ligated As, Sb and Bi compounds towards electron deficient alkynes and maleimides, dmp = 2,6-Me_2_C_6_H_3_.^94,95^

**I** was also shown to react with MeOTf which induced a rapid dimerization of the starting material and the P-methylated intermediate to give bowl shaped **30**, which features five 5-membered rings within its structure (Scheme 14).^92^ DFT computations suggests that the initial methylation proceeds at the phosphorus atom. This species is subsequently trapped by the starting material, despite the predicted thermodynamic preference for methylation in α-position to the nitrogen donor atom. This is in stark contrast with the structural related ONO system **4** that exhibits a non-planar molecular structure and is best described as P(iii). Upon reaction with methyl triflate the nitrogen atom of the pincer scaffold is selectively methylated (**31**, Scheme 14).^85^ In contrast, the reaction with nucleophiles proceeds at the phosphorus atom, indicative of an available acceptor orbital due to the P(iii) electronic configuration. The CCC ligated phosphorus compound **32** (ref. 68) reacts with Mo(CO)_5_(THF) under simple L-type ligation of the metal center.^93^ When the reaction is instead performed with Mo(CO)_6_, insertion of a carbonyl ligand into the P–C bond is observed, accompanied by Mo(CO)_5_ coordination. The Mo(CO)_5_ fragment can be cleaved from the phosphorus ligand upon addition of dppe (Scheme 14). Upon coordination of AlBr_3_ towards the inserted carbonyl group, the adduct **33** is formed which dimerizes in the solid state towards **33-dimer** upon attack of the phosphorus atoms at the α-position to the enolate, giving rise to a P_2_C_22_ scaffold. Constrained pnictogens have also shown great potential as sources for masked pnictinidenes and can engage in Diels–Alder type transformations.^54^ A series of cycloaddition reaction patterns was reported by Dostál for the reaction of T-shaped As, Sb and Bi species towards electron deficient alkynes and maleimides (Scheme 15). **3-As** initially reacts with an electron deficient alkyne to form the Diels–Alder product **34** which rapidly reacts to the 12π-electron aromatic 1-arsanapthalene **35**.^94^ The reaction is stereospecific as it favours the smaller substituent of the alkyne to occupy the 2-position in the final product. When using **3-Sb** as a starting material, the Diels–Alder product **36** proved to be stable and cannot be converted to a conjugated ring system even upon heating (Scheme 15). Furthermore, the reactivity towards maleimides was studied.^95^ The C=C double bonds adds along the Sb–N bond giving the [4 + 2] cycloaddition product **37**. In solution both species are in equilibrium as determined by EXSY NMR studies. DFT study of the reaction mechanism reveals that the HOMO of **3-Sb** which is represented by an Sb p-type lone-pair with partial delocalization over the aromatic back bone overlaps with the maleimide LUMO resulting in a significant drop of C_arene_–Sb bond ellipticity as determined by QTAIM calculations in the reaction products. This suggests a significantly diminished π-interaction upon formation of Sb(iii).^96^ A possible [1 + 2] cycloaddition followed by ring expansion, akin to recent reports for singlet phosphinidenes,^97^ is being ruled out on the basis of theoretical calculations. In the case of the reaction of **3-Bi** with electron deficient alkynes, the reaction outcome heavily depends on the substitution pattern of the utilized alkyne (Scheme 15).^94^

The reaction with dimethylacetylenedicarboxylate produces the Diels–Alder reaction product **38** with no observed further conversion towards naphthalene derivatives. The same product can be obtained when methyl propiolate is used. However, addition of a second equivalent results in cleavage of the Bi–N bond upon proton abstraction from the alkyne to give **39**. This showcases the difference in the polarity of Pn–N bonds within group 15 and the different reaction patterns that can consequently be achieved. Furthermore, **3-Bi** was shown to oxidatively add C(sp^3^)–X bonds to give five-coordinate Bi(iii) species.^98^

## 5. Conclusions

The chemistry of geometrically constrained group 15 compounds has opened up a wide variety of possible chemical transformations. The nature of the supporting ligand allows for tuning of the energetic levels of frontier orbitals, geometry and oxidation state of the central pnictogen atom. In addition to their fascinating reactivity and potential applications in small molecule activation chemistry and catalysis, these compounds are also promising chemically “non-innocent” ligands in transition metal chemistry, allowing for cooperative transformations. Depending on the supporting ligand, Pn-centred or cooperative activation products can be obtained when reacted with polar E–H bonds. Besides stoichiometric bond splitting reactions of challenging substrates such as ammonia or water, which still represent a major challenge for transition metals, even catalytic approaches for hydrogenation and hydroboration were realized. The observed reactions with simple carbon-based substrates can afford unexpected reaction products and show great potential for new main group mediated bond formation reactions. Furthermore, the nature of the utilized pnictogen atoms can induce significant polarity differences to allow for different transformations within an isostructural series.

This thriving field of main group chemistry is far from being well explored and there are hopefully many more unforeseen reaction patterns to be investigated in the next years. Especially the derivatization of the ligand systems to allow for effective turnover of catalytic reactions by flattening the overall potential energy surface^99,100^ represents a major goal for future research.

## Conflicts of interest

There are no conflicts to declare.
